# ANGPTL3 impacts proteinuria and hyperlipidemia in primary nephrotic syndrome

**DOI:** 10.1186/s12944-022-01632-y

**Published:** 2022-04-10

**Authors:** Fu Zhong, Shurao Liu, Yue Li, Guanyu Li, Ming Liu, Jingzhi Wang, Weijing Cui, Yanhong Suo, Xia Gao

**Affiliations:** 1 Nephrology Department, Guangzhou Women and Children’s Medical Center, Guangzhou Medical University, No. 318 Renmin Middle Road, 510623 Guangzhou city, China; 2 Pediatric Intensive Care Unit Department, The First People’s Hospital of Zhaoqing, 526000 Zhaoqing city, China; 3 Guangzhou Institute of Pediatrics, Guangzhou Women and Children’s medical center, 510623 Guangzhou city, China; 4 Pediatric Department, Gansu Provincial Hospital, 730000 Lanzhou City, China

**Keywords:** ANGPTL3, Hyperlipidemia, Proteinuria, Nephrotic syndrome

## Abstract

**Background:**

It is unclear why primary nephrotic syndrome (PNS) patients often have dyslipidemia. Recent studies have shown that angiopoietin-like protein 3 (ANGPTL3) is an important regulator of lipid metabolism. In this study, we explored how ANGPTL3 impacts dyslipidemia during PNS development.

**Methods:**

We measured the serum levels of ANGPTL3 in PNS patients (*n*=196). Furthermore, the degree of proteinuria and lipid metabolism were examined in *angptl3*-overexpressing transgenic (*angptl3*-tg) mice at different ages. Moreover, in this study, we used the clustered regularly interspaced short palindromic repeats-associated protein 9 (CRISPR/Cas9) system to create *angptl3*-knockout (*angptl3*-/-) mice to investigate lipopolysaccharide (LPS)-induced nephrosis.

**Results:**

Compared with that in the healthy group, the serum level of ANGPTL3 in the PNS group was significantly increased (32 (26.35-39.66) ng/ml vs. 70.44 (63.95-76.51) ng/ml, Z =-4.81, *P* < 0.001). There were significant correlations between the serum level of ANGPTL3 and the levels of cholesterol (*r*=0.34, *P* < 0.001), triglycerides (*r*= 0.25, *P* = 0.001) and low-density lipoprotein (*r*= 0.50, *P* < 0.001) in PNS patients. With increasing age, *angptl3*-tg mice exhibited increasingly severe hypertriglyceridemia and proteinuria. The pathological features of *angptl3*-tg mice included rich lipid droplet deposition in hepatocytes and diffuse podocyte effacement. Compared to wild-type mice, *angptl3*-/- mice showed significantly lower degrees of lipid dysfunction and proteinuria after stimulation with LPS. The effects of ANGPTL3 on nephrotic dyslipidemia were confirmed in cultured hepatocytes subjected to *angptl3* knockdown or overexpression. Finally, significant alterations in lipoprotein lipase (LPL) levels were observed in liver tissues from Angptl3-/- and wild-type mice stimulated with LPS.

**Conclusions:**

ANGPTL3 could be involved in the development of dyslipidemia, as well as proteinuria, during PNS pathogenesis. Inhibition of LPL expression may the mechanism by which ANGPTL3 induces hyperlipidemia in PNS.

**Supplementary Information:**

The online version contains supplementary material available at 10.1186/s12944-022-01632-y.

## Introduction

Proteinuria and hyperlipidemia are considered to be the most important clinical features of primary nephrotic syndrome (PNS). Among them, proteinuria is the core characteristic of PNS. Podocyte injury in the outermost layer of the glomerular filtration barrier plays a key role in the development of PNS proteinuria [1]. Hyperlipidemia, which often appears during the acute phase and disappears in the remission phase of the disease, is another important feature of PNS. Abnormal lipoprotein lipase (LPL) activity is considered to be one of the mechanisms leading to PNS hyperlipidemia [2]. However, why PNS results in massive proteinuria and hyperlipidemia at the same time and whether these two pathophysiological phenomena have a common pathogenic mechanism are questions of interest.

Angiopoietin-like protein 3 (ANGPTL3) belongs to the angiopoietin-like protein family. ANGPTL3 is mainly synthesized by liver cells and is notably expressed in kidney podocytes [3]. Recently, a series of experiments showed that ANGPTL3 could induce cytoskeletal rearrangement in podocytes, leading to increased podocyte motility [4]. Some studies have revealed that ANGPTL3 plays a role in the development of nephrotic proteinuria by attenuating podocyte foot effacement and podocyte detachment from glomeruli [5].

As a lipid regulating reagent, ANGPTL3 has been extensively studied with respect to lipid metabolism [6]. ANGPTL3 can significantly inhibit the activity of LPL, leading to reduced triglyceride and cholesterol decomposition and increased blood lipid levels, and is a key molecule that regulates lipid metabolism [7]. A study on ANGPTL3 plasma levels and extracoronary arterial health showed that the ankle–brachial blood pressure index was significantly associated with ANGPTL3 levels [8].

In this study, we examined whether ANGPTL3 could regulate lipid metabolism in vivo or in vitro in PNS models, and by using *angptl3*-tg mice, we examined the multiple effects of ANGPTL3. Finally, we explored the role of LPL in the mechanism of ANGPTL3 in PNS hyperlipidemia.

## Methods

### Antibodies and reagents

The antibodies and reagents used in this study are listed with their sources in parentheses: a monoclonal antibody against glyceraldehyde-phosphate dehydrogenase (GAPDH) (ImmunoWay Biotechnology, Texas, USA), a polyclonal antibody against ANGPTL3 (R&D Systems, Minneapolis, USA), and a polyclonal antibody against LPL (Santa Cruz Biotechnology, Santa Cruz, Santa Cruz, CA, USA). Lipopolysaccharide (LPS) was purchased from Pfizer Inc., New York City, NY, USA.

### Objectives

In this study, 196 patients with PNS from China admitted to Gansu Province People’s Hospital from Jan 2016 to Jan 2018 were enrolled, including 124 males and 72 females. The study protocol conformed to the ethical guidelines of the 2013 Declaration of Helsinki. We ensured that all patients and healthy controls (HCs) provided written informed consent for the study, and ethics approval was obtained from the Gansu Province People’s Hospital Research Ethics Committee (syll 20,160,037).

The PNS patient inclusion criteria were as follows: heavy proteinuria [24-hour urine total protein (24 hUTP) > 3.5 g, urine protein/creatinine ratio > 3.0 mg/mg, or 24 hUTP > 50 mg/kg] and hypoalbuminemia [serum albumin (ALB) ≦25 g/L], along with various degrees of edema and hyperlipidemia [9, 10].

The HCs had no concomitant health problems, and fasting blood lipid levels [10] [cholesterol (CHOL) < 200 mg/dl, triglycerides (TG) < 150 mg/dl, and low-density lipoprotein cholesterol (LDL-C) < 130 mg/dl] and urinary proteins (urinary microalbumin ≤ 150 mg/dl or negative urine qualitative test) were within the normal range.

The exclusion criteria were as follows: PNS patients without the required clinical and laboratory data, individuals without with secondary nephrotic syndrome, individuals without a previous history of other acute or chronic kidney disease, patients with abnormal ultrasound examination of the urinary system (e.g., deformities, cysts, hydrops, or stones), patients with an identified acute or chronic illness (diabetes mellitus, thyroid dysfunction, polycystic ovary syndrome, obesity, fatty liver, or familial hypercholesterolemia), and patients with other systemic diseases, such as hematological diseases, cardiovascular diseases, connective tissue diseases, tumors, and obvious infections.

### Experimental methods

(1) Sample collection: Elbow venous blood (3 ml) was collected from fasting subjects in the morning in an anticoagulant test tube. Serum was separated after centrifugation for 5 min (800 x g) and transferred to EP tubes. Five milliliters of urine were collected and placed in a test tube without any additives. After centrifugation for 5 min (800 x g), the supernatant was collected and transferred into EP tubes. Serum and urine were frozen at -80 °C for use.

(2) Detection of serum and urine biochemical indicators: Biochemical indicators such as triglycerides, total cholesterol (TC), high density lipoprotein (HDL-C), LDL-C, serum creatinine (Scr), urea nitrogen (BUN), and 24-hour urea protein (24 hUP) were measured with an automatic biochemical analyzer (Abbott ARCHITECT c1600, USA).
(3) Determination of ANGPTL3 levels in serum: ANGPTL3 concentration in serum samples was measured using the ELISA kit of human ANGPTL3 from Jiangsu MEIMIAN Ltd (Yancheng City, China). The lowest detection limit of this kit was 2 ng/ml.

### Preparation of Cas9 mRNA and sgRNA

The following 20-nucleotide guide target sequence was selected within the first 200 nt from the start codon of the Angptl3 gene: GAGTGGATCCAGACCTTTCA. The target sequence was cloned into the pX330-U6-Chimeric_BB-CBh-hSpCas9 vector (Addgene_42230) as previously described. Next, Cas9 CDS and sgRNA sequences were PCR amplified from the vector backbone with the T7 promoter sequence added to the 5’ end of the forward primer. The primer sequences are provided in Table S1. The T7-Cas9 and T7-sgRNA PCR products were gel purified with a QIAquick Gel Extraction Kit (Qiagen, USA) and used as templates for in vitro transcription (IVT) by the mMESSAGE mMACHINE™ T7 Transcription Kit (Thermo Fisher Scientific, USA) and the MEGAshortscript™ T7 Transcription Kit (Thermo Fisher Scientific, USA), respectively. Both Cas9 mRNA and sgRNA were purified according to standard protocols for phenol:chloroform extraction and ethanol precipitation and were then dissolved in DNase/RNase-free water (Thermo Fisher Scientific, USA).

### Generation of Angptl3-knockout mice

Female C57BL/6 mice (6–8 weeks old) were used as embryo donors. Female C57BL/6 mice were superovulated by intraperitoneal injection with pregnant mare serum gonadotropin (PMSG) and human chorionic gonadotropin (hCG) and then mated with male C57BL/6 mice. Fertilized embryos (zygotes) were collected from the oviducts. Cas9 mRNA (100 ng/µL) and sgRNA (*angptl3*) (50 ng/µL) were mixed and injected into the cytoplasm of fertilized eggs with both pronuclei visible in Chatot–Ziomek–Bavister (CZB) medium. The injected zygotes were then cultured in Quinn’s Advantage cleavage medium (In Vitro Fertilization, Inc.) at 37 °C and 5% CO_2_ for approximately 24 h, and 18–20 2-cell stage embryos were transferred into the oviduct of a pseudopregnant ICR female mouse at 0.5 dpc. The F0 mice were genotyped using *angptl3* primers (F and R) (Table S2). T-clone and Sanger sequencing were performed to identify the Angptl3 KO F0 mice with the desired frameshift mutation. F0 mice were further crossed with C57 mice to obtain Angptl3 +/- F1 mice. Homozygous KO mice were obtained by breeding heterozygous KO mice. This work was performed at Shanghai Gemple Biotech Co., Ltd.

### Mouse identification and maintenance

All mice had access to food and water. All experiments were performed in accordance with the Health Guide for the Care and Use of Laboratory Animals and were approved by the Biological Research Ethics Committee of Gansu Province People’s Hospital (No. syll20130331). Genotyping of *angptl3*-/- mice was performed by PCR analysis of mouse tail-tip genomic DNA using *angptl3* primers (F and R) (Table S2) and then analyzed by Sanger sequencing. This work was performed in the Animal Center of Gansu University of Traditional Chinese Medicine. All mice were housed in an air-conditioned room and were provided free access to food and water (22 ± 2 °C; 12:12-hour light:dark cycle). After the mice were anesthetized with 10% chloral hydrate (400 mg/kg), they were euthanized by cervical dislocation, and all efforts were made to minimize pain and discomfort. The mice did not exhibit signs of peritonitis after the administration of 10% chloral hydrate (400 mg/kg).

### Generation of LPS nephrosis in mice

All animal studies were approved by the Subcommittee on Research Animal Care of the Gansu Province People’s Hospital (No. syll20130331) and performed at the Animal Center of Gansu University of Traditional Chinese Medicine. Thirty-six male wild-type or Angptl3-/- C57BL/6 mice aged 6 to 8 weeks old were given free access to standard laboratory food and water. Both groups of mice were injected intraperitoneally with 200 µg of LPS (1 mg/ml in sterile LPS-free PBS) in a total volume of 200 µl. Mice in the control group (*n*=5) were intravenously administered an identical volume of saline. After the four groups were injected, urinary protein excretion was measured at 24 h, 48 h, and 72 h, and kidney and liver tissues were harvested and processed for H&E staining. FP effacement was assessed by transmission electron microscopy according to our published protocols [11]. After LPS injection, the mice were killed at 24 h, 48 h, and 72 h, during which time no unexpected deaths were observed. The humane endpoint was defined as weight loss of 20%, dyspnea, or difficulty feeding within 72 h of LPS injection. Death was confirmed by the absence of a pulse, breathing, a corneal reflex, or a response to toe pinching and a lack of respiratory sounds and heartbeat.

### Generation of *angptl3* transgenic mice

Murine *angptl3* cDNA was synthesized by Shanghai Gemple Biotechnology and cloned into the pcDNA3.1 vector (Fig. S2a). This plasmid, designated pcDNA3.1-Angptl3, was linearized by MluI/DraIII, and the fragment of interest was then purified for oocyte injection into C57Bl/6 mouse-derived fertilized eggs [12, 13]. Transgenic mice were identified by PCR using oligonucleotide primers specific for the construct (CMV-F, 5’-CGCGTTGACATTGATTATTGA CTA -3’ and *angptl3*-R, 5’- CAGGAGGCCATTCG CTAAAA -3’; PCR fragment =892 bp) (Fig. S2b).

### Hepatocyte cell line culture and treatment

A nontumorigenic mouse hepatocyte cell line (AML12) was obtained from the American Type Culture Collection (ATCC) and cultured in DMEM/F12 medium (Gibco) containing 5 µg/ml ITS premix (Sigma–Aldrich, USA), 40 ng/ml dexamethasone (Sigma–Aldrich), and 10% fetal bovine serum (FBS, Gibco) at 37 °C in a humidified atmosphere with 5% CO2. Lipopolysaccharide (LPS; working concentration: 25 µg/ml) was purchased from Sigma–Aldrich. AML12 cells were collected after LPS stimulation for 24 h.

### Lentiviral infection

To produce ANGPTL3 overexpression or knockdown lentivirus, lentivirus with the murine angptl3 coding sequence or angptl3 shRNA and blank controls were purchased from Gemple Biotechnology (Shanghai, China). The target sequences of the angptl3 shRNA were as follows: sh angptl3#1, 5’-GCTGGG TCATGGACTTAAAG-3’; and sh angptl3#2, 5’-GCAGCTAACCAACTTAA TTC-3’. AML12 cells were infected with recombinant lentivirus plus 8 µg/ml polybrene (Sigma–Aldrich) at a multiplicity of infection (MOI) of 20. Stable lentivirus-infected cells were selected and enriched by flow cytometry (BD).

### RNA extraction and quantitative RT–PCR

Total RNA was extracted using a Direct-zol RNA MiniPrep kit (Zymo Research, USA) according to the manufacturer’s instructions. Total mRNA (1 µg) was reverse transcribed using 5X All-In-One RT MasterMix (Abm, Canada) according to the manufacturer’s instructions. Real-time PCR (RT–PCR) was performed using SYBR FAST qPCR Kit Master Mix (2X) Universal (KAPA, USA) on an Applied Biosystems 7,500 Fast RT–PCR System (Foster City, USA). The RT–PCR mixture included cDNA (1.0 µl), 2X SYBR‑Green Mix (10 µl), forward primer (10 µM, 0.5 µl), reverse primer (10 µM, 0.5 µl), and RNase‑free water in a final volume of 20 µl. The reaction conditions were as follows: 2 min of denaturation at 94°C, 40 cycles of 1 min at 94°C, 30 sec at 56°C, and 2 min at 72°C, and a final extension step at 72°C for 10 min. The cycle threshold (Ct) values were analyzed using the comparative Ct (ΔΔCt) method according to the MIQE guidelines. The amount of the target was normalized to an endogenous reference (GAPDH) and is expressed relative to the control (nontreated cells). The primers used were as follows: ANGPTL3 (forward, 5’- GCGAACATACAAGTGGCGTG-3’; reverse, 5’-CTGTGAGCCATCT TTCCGGT-3’) and LPL (forward, 5’- GAAAACCCCAGC AAGGCATAC -3’; reverse, 5’- CATCTTGCTGCTTCTCTTGGC -3’).

### Oil Red O staining

Oil Red O staining was performed with an Oil Red O Stain Kit (Jiancheng Bioengineering Institute, Nanjing, China) according to the manufacturer’s instructions. The results were examined with a light microscope, and the OD560 was measured for quantification.

### Western blotting

We performed immunoblotting experiments as described previously [4]. The issue to be tested in radioimmunoprecipitation assay (RIPA) buffer and centrifuged at 12,000 rpm for 15 min at 4 ℃. The supernatant was collected and the total protein content was determined by bicinchoninic acid (BCA) Protein Assay Kit (Solarbio). The protein was separated on sodium dodecyl sulfate-polyacrylamide gel electrophoresis (SDS-PAGE) gel and transferred to polyvinylidene fluoride (PVDF) membrane. The membrane was sealed with Blocking Buffer (Biosharp) for 30 min. the membranes were incubated with primary antibodies at appropriate dilutions in PBS/Tween (PBST) overnight at 4 ℃. subsequently, after washing with PBS/Tween (PBST) for 3 × 5 min, the membrane was incubated with secondary antibody at 37 ℃ for 2 h. After washing, the results were visualized using an enhanced chemiluminescence system (Bio Rad).

### Statistical analysis

The experimental data were tested for normality using the Kolmogorov–Smirnov method, and the chi-square test was performed using Levene’s test for variance equations. All quantitative data conforming to a normal distribution are expressed as the x±s and were analyzed using independent-sample t tests; nonnormally distributed quantitative data are expressed as the median and 95%CI and were analyzed using the Mann–Whitney U test. Differences in sex ratios were tested by the *X*^2^ test. Pearson correlation analysis was used to examine correlations between variables of two measures that had a normal distribution; the Spearman rank correlation test was used to analyze correlations between variables of two measures that did not have a normal distribution. The values of animals or cells were subjected to one-way ANOVA, and Pearson correlations among the groups were calculated. *P* values of <0.05 were considered statistically significant. The data were statistically analyzed using SPSS 20.0 software.

## Results

### The serum level of ANGPTL3 correlated with blood lipids in PNS patients

This study included 196 patients with PNS, with 72 females (36.54%), 124 males (63.46%), and a male-to-female ratio of 1:0.58. The average age of the PNS group was 32 years (32.88-37.75) and that of the healthy control group was 31.5 years (29.37-42.83); there was no significant difference between the two groups (*P* > 0.05), as shown in Table 1.

**Table 1:** Comparison of basic characteristics between the both groups [M(95%CI),n(%)]

	Health Group (*n*=60)	PNS (*n*=196)	Z/x^2^	*P*
Male /female	30/30	124/72	1.79	0.18
Ages (years)	31.5(29.37-42.83)	32(32.88-37.75)	-0.37	0.71
CHO (mmol/L)	3.88(3.26-4.13)	6.53(6.53-7.50)	-5.58	<0.001
TG (mmol/L)	0.95(0.83-1.25)	2.05(2.18-2.69)	-5.34	<0.001
LDL (mmol/L)	1.96(1.69-2.15)	3.96(4.10-4.85)	-5.97	<0.001
HDL (mmol/L)	1.22(1.03-1.32)	1.65(1.77-2.06)	-4.36	<0.001
24hUP(g/d)	0.09(0.08-1.00)	1.75(2.26-3.98)	-5.27	<0.001
ALB(g/L)	45.4(41.01-46.69)	31.05(30.05-33.15)	-5.21	<0.001
Scr(umol/L)	55.2(41.81-58.41)	72.45(74.53-94.07)	-3.93	<0.001
BUN(mmol/L)	5.45(4.61-6.08)	6.36(4.66-14.22)	-2.12	0.34
Serum ANGPTL3(ng/ml)	32(26.35-39.66)	70.44(63.95-76.51)	-4.81	<0.001

Note: *CHO* cholesterol, *TG* triglyceride, *LDL* low density lipoprotein, *HDL* high density lipoprotein.

This study analyzed the serum level of ANGPTL3. Compared with that in the healthy group, the serum level of ANGPTL3 in the PNS group was significantly increased (32 (26.35-39.66) ng/ml vs. 70.44 (63.95-76.51) ng/ml, Z =-4.81, *P* < 0.001). Furthermore, we analyzed the correlations between serum ANGPTL3 levels and major indicators of blood lipids and found that ANGPTL3 positively correlated with CHO to a low degree (*r*=0.34, *P* < 0.001), with triglycerides to a low degree (*r*= 0.25, *P* = 0.001), and with LDL-C to a moderate degree (*r*= 0.50, *P* < 0.001), but there was no correlation with HDL-C (Table 2, *r*= 0.15, *P* = 0.07).

**Table 2:** The correlation between serum ANGPTI3 and lipid

Factor	Serum ANGPTL3	
	r/r_s_	*P*
CHO	0.34	<0.001
TG	0.25	0.001
LDL	0.50	<0.001
HDL	0.15	0.07

Note: *CHO* cholesterol, *TG* triglyceride, *LDL* low density lipoprotein, *HDL* high density lipoprotein.

### Under physiological conditions, lipid and proteinuria levels in *angptl3*-knockout C57 mice were nearly normal

**Fig. 1:**
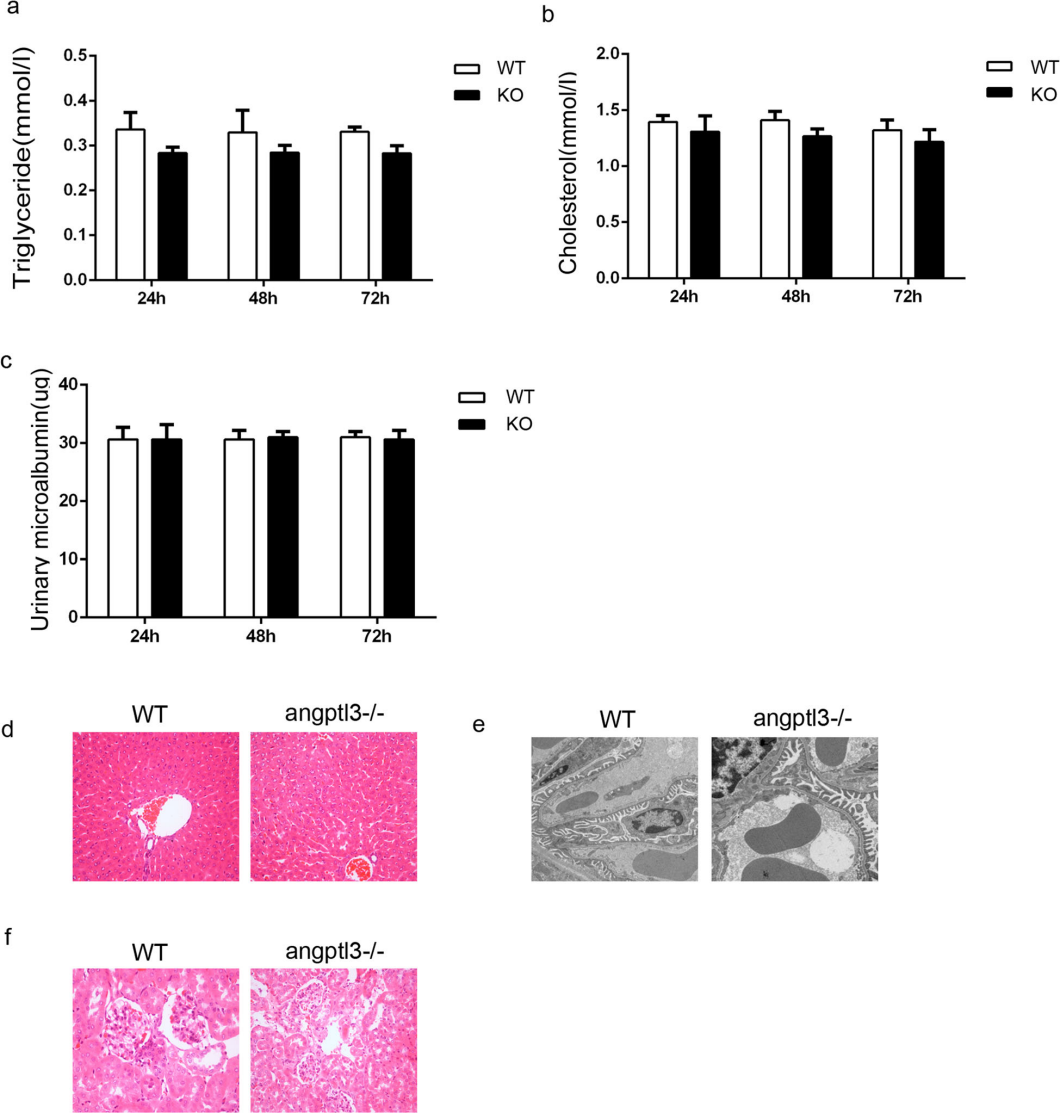
Under physiological conditions, the lipid and protein levels in *angptl3*-knockout C57 mice were nearly normal. **a, b, c**: The levels of triglycerides, cholesterolemia and proteinuria were not different between the groups. (*P* > 0.05). **d**: Under a light microscope, there were no changes in the liver structure between wild-type and angptl3-/- mice. **e, f**: The glomerular structure of knockout mice was also normal by light and electron microscopy. WT: wild type mice, KO: Angptl3-/- mice. *: *P*<0.05, **: *P*<0.01; the *P* values were derived from independent-sample t tests

To explore the function of *Angptl3* gene, we build the *angptl3*-/- mice by CRISP-CAS9 tech as supported by the sanger sequencing (The data was showed in supplementary results). We observed the laboratory results and pathological features of *angptl3*-/- mice under physiological conditions. There was no significant difference in serum triglycerides or total cholesterol between wild-type mice and *angptl3*-/- mice (Fig. 1a, b *P* > 0.05). The 24 h urine protein results showed that there was no difference between *angptl3*-/- mice and wild-type mice (Fig. 1c, *P*> 0.05). The liver structure of *angptl3*-/- mice was observed under a light microscope, and there was no difference between wild-type and *angptl3*-/- mice. No inflammatory cell infiltration was observed (Fig. 1d). The glomerular structure of knockout mice was also normal by light and electron microscopy (Fig. 1e, f).

### In an LPS-induced nephrosis mouse model, *angptl3* knockout in C57 mice alleviated proteinuria and hyperlipidemia

**Fig. 2:**
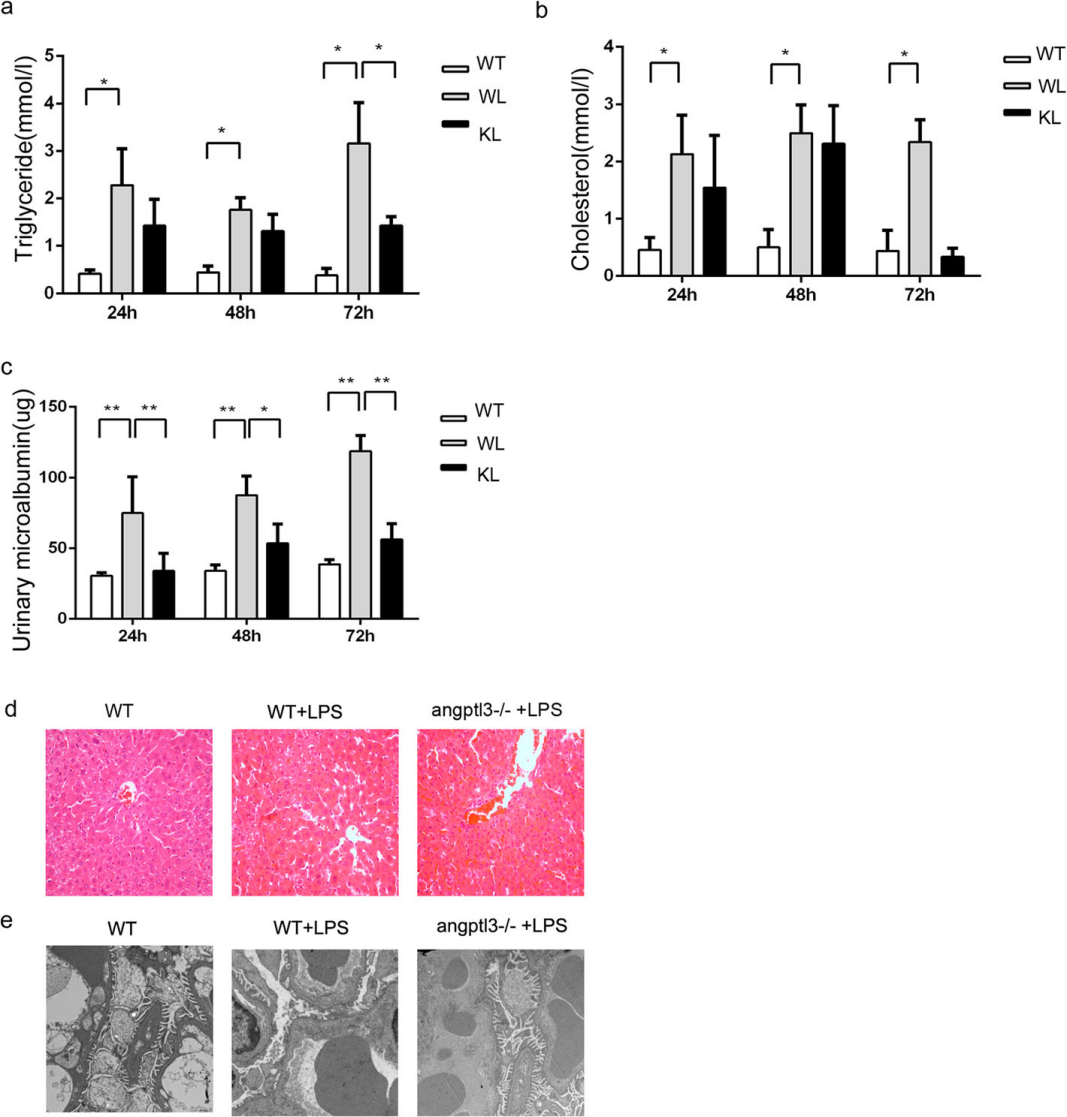
Knockout of the angptl3 gene did not affect the liver or kidney function of mice in the physiological state and played an important role in LPS-induced nephropathy. **a, b, c**: The levels of triglycerides, cholesterolemia and proteinuria in the three groups. **d**: Lipid droplet deposition in the liver tissue of each group of mice, with a magnification of 400X. **e**: Changes in the different groups, with a magnification of 5,000X. WT: wild-type mice, WL: wild-type mice stimulated with LPS, KL: Angptl3-/- mice stimulated with LPS. Each group included 5 mice. *: *P*<0.05, **: *P*<0.01; the *P* values were derived from one-way ANOVA

To explore whether ANGPLT3 impacts dyslipidemia under PNS conditions, we compared changes in the main indices of blood lipids in wild-type and *angptl3*-/- mice after LPS treatment. As shown in Fig. 2a and b, compared with those in the WT group, mice in the WT+LPS group developed significant hypertriglyceridemia and hypercholesterolemia after 48 h (*P* < 0.05). The triglyceride and total cholesterol levels in the *angptl3*-/-+LPS group were lower than those in the WT+LPS group at 24 and 48 h, and a significant difference was observed at 72 h (*P* < 0.05).

Then, we observed lipid droplet deposition in the liver tissues in each group of mice at 72 h. The results showed liver cell vacuolar degeneration in the WT+LPS group compared with the WT group, indicating that lipid droplets accumulated in large amounts in hepatocytes (Fig. 2d**)**. In contrast to that of wild-type mice, lipid droplet deposition in the liver tissues of *angptl3*-/- mice showed a significant reduction after LPS stimulation.

In this study, we also compared the changes in proteinuria in *angptl3*-/- mice and wild-type mice in an LPS-induced nephropathy model. As shown in Fig. 2c, at 24, 48 and 72 h after LPS induction, the proteinuria levels of *angptl3*-/- mice at the different time points were significantly lower than those of wild-type mice (*P* < 0.05).

The structure of glomeruli and podocytes was observed and was consistent with the pathological phenotype of MCD, and the structure of glomeruli in wild-type mice after LPS stimulation was mainly normal under a light microscope. The podocytes of mice with LPS-induced nephropathy were extensively fused when viewed under an electron microscope. However, the degree of podocyte fusion in *angptl3*-/- mice was significantly lower than that in wild-type mice after LPS stimulation (Fig. 2e).

### Transgenic *angptl3* mice developed hyperlipidemia accompanied by proteinuria

**Fig. 3:**
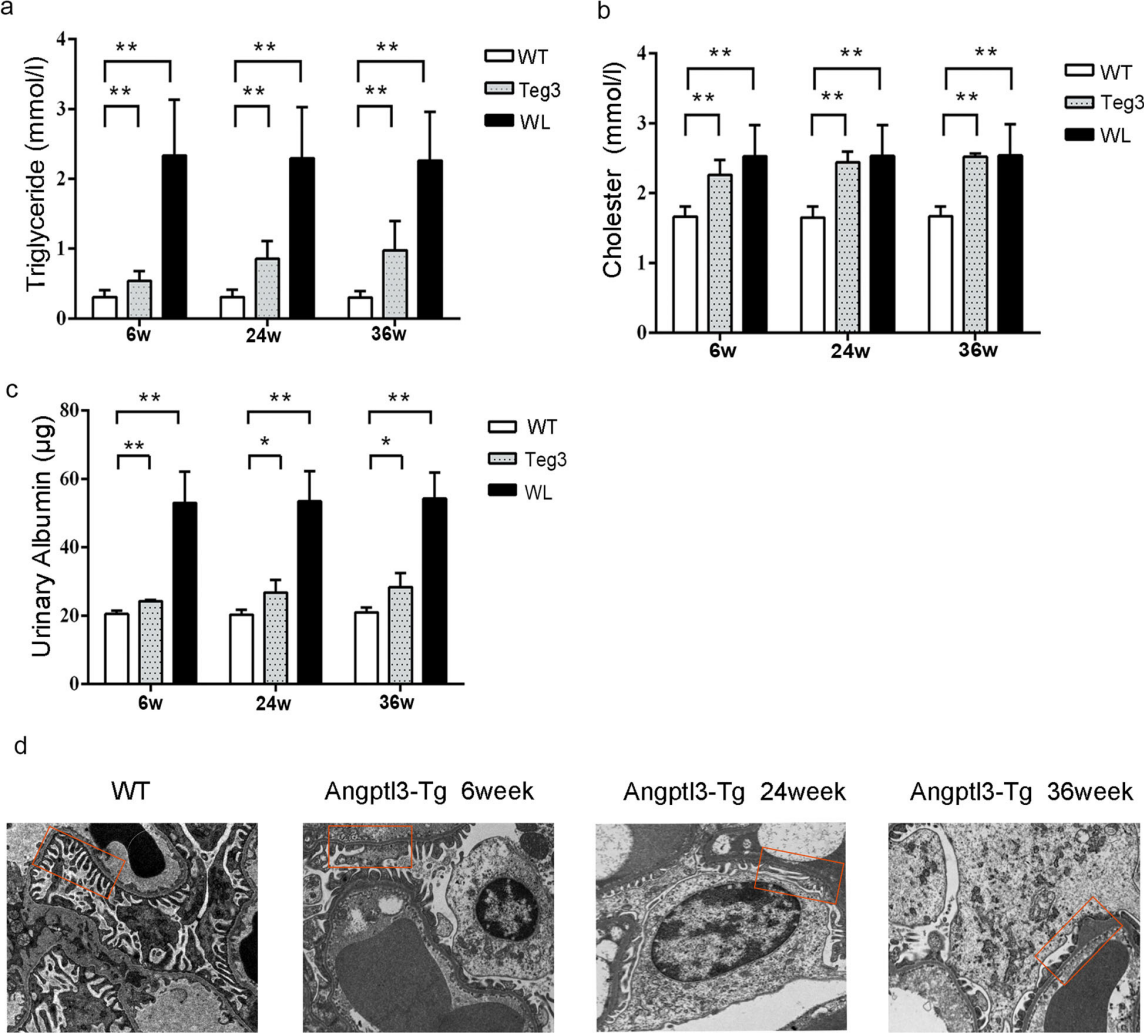
Angptl3-tg mice developed hyperlipidemia and varying degrees of proteinuria. **a**: Triglyceride levels in angptl3-tg mice were markedly increased at different time points. **b**: The cholesterol level in angptl3-tg mice was also significantly higher than that in wild-type mice at every time point. **c**: Changes in proteinuria levels in the three groups. **d**: Random EM images of the glomerular basement membrane surrounded by the epithelium and endothelium were taken at a magnification of 5,000X. WT: wild-type mice, WL: wild-type mice with LPS stimulation. Each group included 5 mice. *: *P*<0.05, **: *P*<0.01; the *P* values were derived from one-way ANOVA

To verify whether ANGPTL3 is involved in both lipid metabolism and proteinuria, angptl3-transgenic mice were examined. As shown in Fig. 3a, b, the triglyceride and total cholesterol levels of *angptl3*-tg mice were significantly higher than those of wild-type mice at the three time points (*P* < 0.01), and the serum lipid index of angptl3-tg mice gradually increased beginning 6 weeks after birth. Furthermore, we investigated possible differences in the degree of dyslipidemia between the LPS nephrotic model and *angptl3*-tg mice. Wild-type mice at 6, 24 and 36 weeks of age were stimulated with LPS. The triglyceride and total cholesterol levels of LPS-stimulated mice were measured after 72 h of LPS stimulation. Furthermore, the triglyceride levels of *angptl3*-tg mice were significantly lower than those of LPS mice at the same age (Fig. 3a, *P* <0.01). In contrast to the triglyceride level, the total cholesterol level was not obviously different between *angptl3*-tg mice and LPS model mice at each age.

In addition, compared with those of wild-type mice, the 24-hour proteinuria quantification results of *angptl3*-tg mice suggested that proteinuria levels increased significantly at week 6 (Fig. 3c, *P* < 0.01). After week 24, the increasing trend was decreased, but the level of proteinuria was still significantly higher than that of wild-type mice of the same age (*P* < 0.05). The 24-hour proteinuria level in the *angptl3*-tg group was significantly lower than that in the wild-type group at each time point after model establishment (*P* < 0.01). In addition, podocyte injury in *angptl3*-tg mice was observed by electron microscopy, and the results showed that podocyte foot effacement became increasingly diffuse with increasing age (Fig. 3d).

### ANGPTL3 regulated lipid metabolism in nephrotic hepatocytes in vitro

**Fig. 4:**
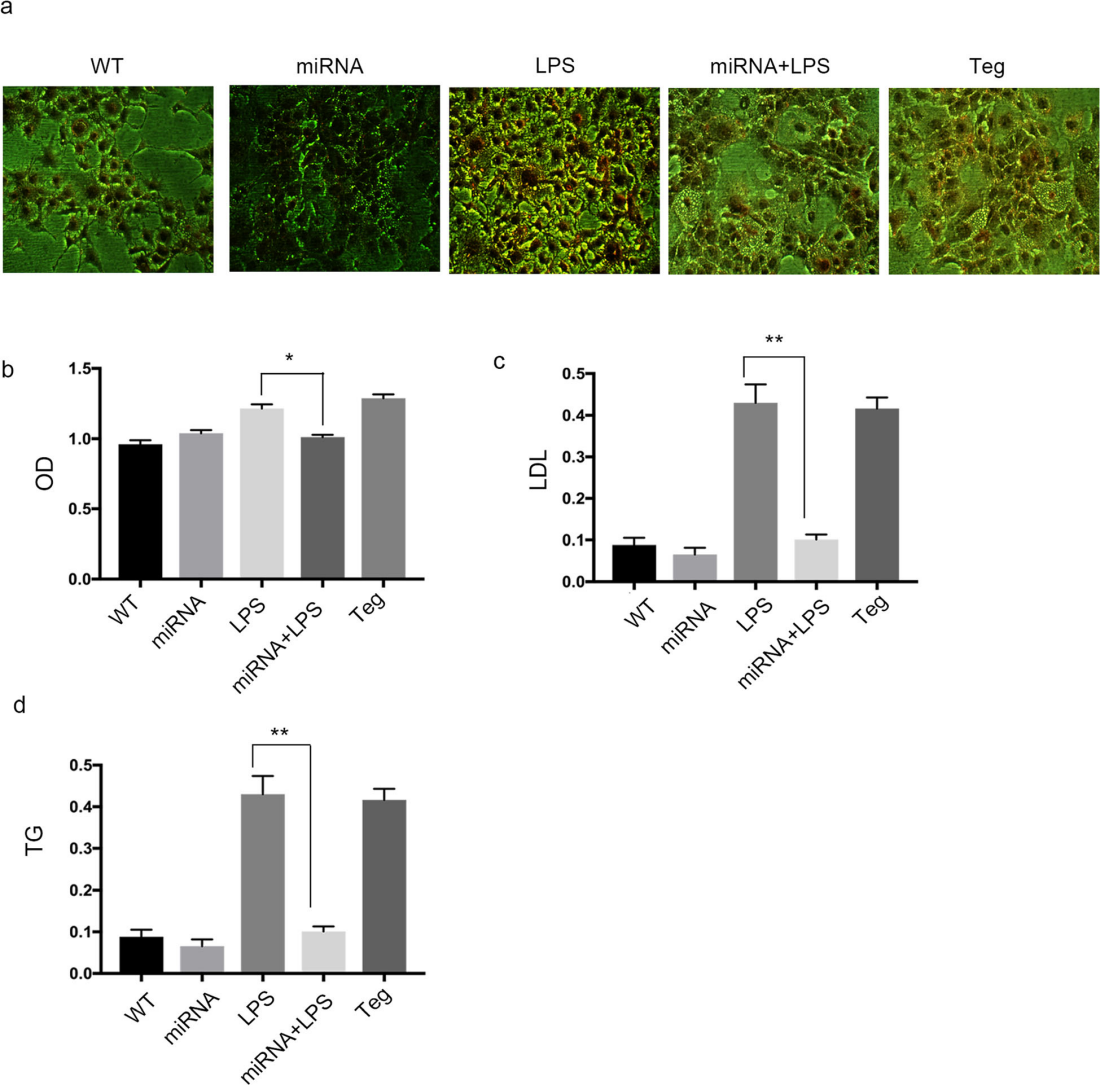
The effects of ANGPTL3 on hepatocytes in the nephrotic state were observed in vitro.** a**: Oil Red O staining areas in the different groups, with a magnification of 400X. **b**: The OD data from Oil Red O staining. **c**: ELISA data showing LDL in the different groups. **d**: Changes in the triglyceride level in the different groups. WT: wild-type hepatocytes, miRNA: angptl3 gene-knockdown hepatocytes, miRNA+LPS: hepatocytes stimulated with LPS after angptl3 knockdown, Teg: hepatocytes transfected with *angptl3*. Each group included 5 mice. *: *P* <0.05, **: *P* <0.01; the *P* values were derived from one-way ANOVA

In this study, lipid metabolism in nephrotic hepatocytes was observed by Oil Red O staining. The results showed that the staining area of wild-type hepatocytes stimulated with LPS was significantly increased compared with that of the untreated group (Fig. 4a, b). The Oil Red O staining area in hepatocytes overexpressing ANGPTL3 was enlarged (Fig. 4a, b). The area of lipid droplets in ANGPTL3-knockdown hepatocytes was significantly smaller than that in wild-type hepatocytes, and both cell lines were stimulated with LPS (Fig. 4a, b).

The triglyceride and LDL levels in each group were further measured by ELISA, as shown in Fig. 4c, d. Wild-type and ANGPTL3-knockdown hepatocytes were stimulated with LPS. We found that the levels of triglyceride and LDL in the miRNA+LPS group were significantly lower than those in the WT+LPS group. The triglyceride and LDL levels in Teg podocytes overexpressing ANGPTL3 were higher than those in the WT group.

### ANGPTL3 affected the occurrence of PNS hyperlipidemia by influencing LPL

**Fig. 5:**
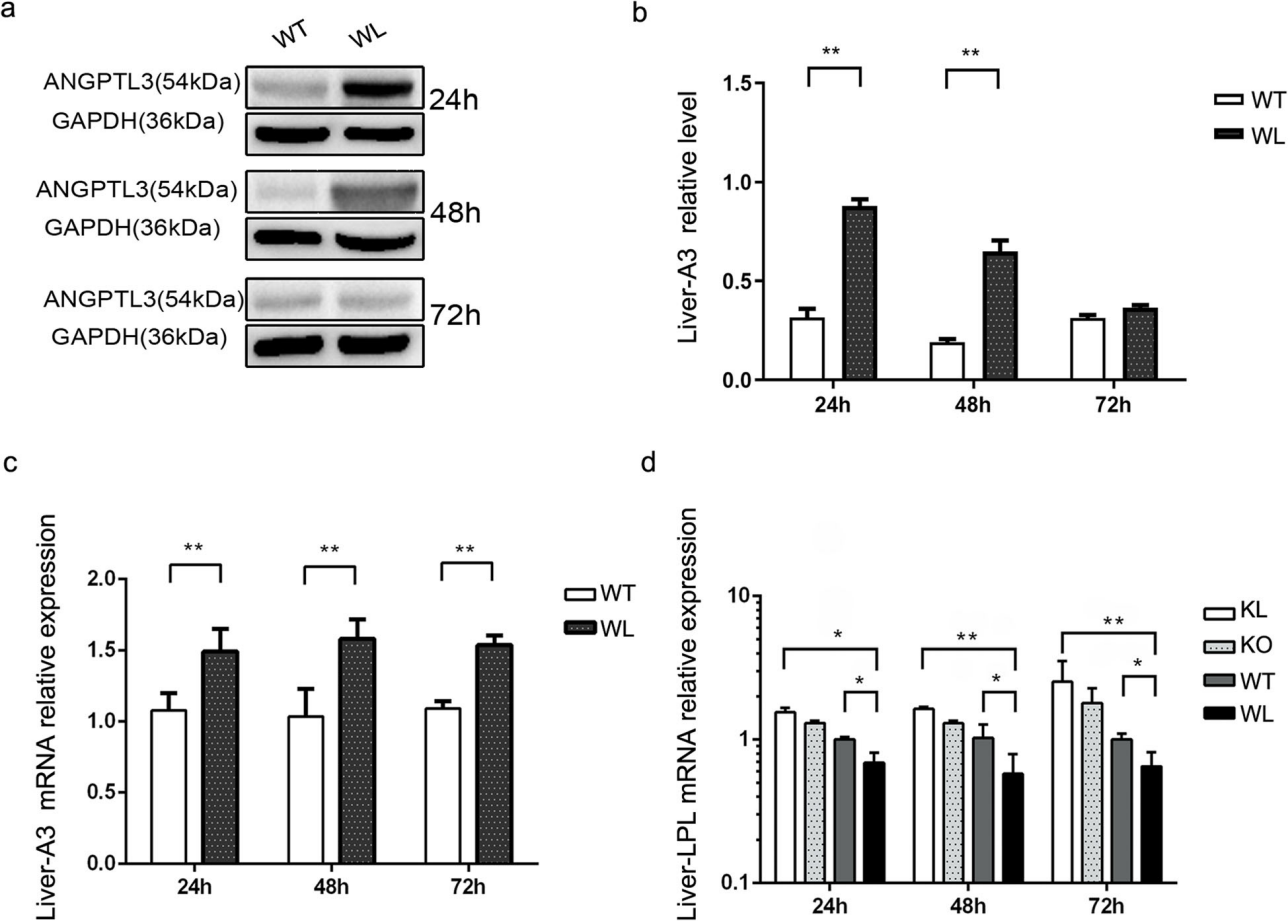
ANGPTL3 may affect the occurrence of PNS hyperlipidemia by impacting LPL expression in mouse liver tissue.: **a, b, c**: ANGPTL3 expression changes in wild-type or Angptl3-/- mice in the physiological and nephritis states. **d** The expression of LPL in wild-type or Angptl3-/- mice in the physiological and nephritis states. WT: wild-type mice, WL: wild-type mice stimulated with LPS, KO: Angptl3-/- mice, KL: Angptl3-/- mice stimulated with LPS. Each group included 5 mice. *: *P*<0.05, **: *P*<0.01; the *P* values were derived from one-way ANOVA

In this study, we measured the expression of ANGPLT3 in liver tissue and found that the mRNA and protein expression levels of ANGPTL3 in the livers of wild-type mice were significantly enhanced 24 h after LPS stimulation **(**Fig. 5a, b, c, *P* < 0.01). ANGPTL3 robustly inhibits the activity of LPL, which is an important factor associated with triglyceride and cholesterol metabolism. Real-time PCR was used to measure LPL mRNA in the liver tissues of mice stimulated with LPS for 24 h, 48 and 72 h. The data showed that the mRNA levels of LPL in the WT+LPS mice were significantly lower than those in the WT group at each time point (Fig. 5d, *P* < 0.05). However, the expression level of LPL in the Angptl3-/-+LPS group was significantly higher than that in the WT+LPS group (Fig. 5d, *P* < 0.05).

## Discussion

The mechanism of massive proteinuria and hyperlipidemia in primary nephrotic syndrome has not been clearly explained. Over the past 10 years, a large number of important signaling molecules and mechanisms related to proteinuria have been identified in the context of podocyte injury [14–16], but the mechanism of PNS complicated with hyperlipidemia has rarely been reported.

As a member of the angiopoietin-like protein family, ANGPTL3 is well known as a powerful regulator of lipid metabolism that inhibits LPL function. Current studies have shown that ANGPTL3 can significantly inhibit LPL activity, thus reducing the rate of triglyceride clearance and increasing plasma triglyceride levels. ANGPTL3 can significantly inhibit the phospholipase activity of endothelial lipase, resulting in reduced plasma HDL-protein hydrolysis [17]. ANGPTL3 can specifically bind to adipocytes and promote the release of fatty acids and glycerol from adipocytes to the liver, where they are further converted into triglycerides and glucose, resulting in an increase in the content of free fatty acids in the plasma [4, 18]. Recent studies have also revealed that ANGPTL3 and ANGPTL8 interact synergistically to significantly enhance the inhibition of LPL activity [19]. ANGPTL3 and ANGPTL8 are essential for efficient storage of dietary triglycerides, and disruption of these genes increases feeding-induced thermogenesis and energy utilization [20]. However, there have been very few studies about this factor in PNS dyslipidemia.

Recently, increasing evidence has suggested that ANGPTL3 is involved in the occurrence of nephropathy-associated proteinuria [4, 11, 21]. In our study, we explored the dual roles of ANGPTL3, which is involved not only in the occurrence of PNS proteinuria but also in hyperlipidemia. First, the data from PNS patients confirmed that serum ANGPTL3 levels could be significantly increased. Furthermore, the degree of hyperlipidemia, as indicated by triglyceride, CHO and LDL-C levels, was correlated with the patients’ ANGPTL3 levels. ANGPTL3 was significantly overexpressed in the serum of PNS patients, suggesting that this molecule may be involved in the occurrence of nephrotic hyperlipidemia. In particular, the correlation between the level of AGNPTL3 and the patient’s lipid metabolism index also suggests that ANGPTL3 plays a role in the pathogenesis of nephrotic hyperlipidemia.

A current report declared that the crystal structure of the fibrinogen-like domains of ANGPTL3 strongly linked to cardiovascular disease [22]. Recent studies suggest that Lp(a) is an independent risk factor for cardiovascular disease, including recurrent cardiovascular events with premature coronary artery disease [23, 24]. It’s known that LPL play a major role in determining the level and composition of plasma Lipoprotein(a) [Lp(a)] [25] and ANGPTL3 could markedly inhibit LPL activity. We think it is very interesting to explore how Lp(a) take part in cardiovascular events with PNS patients and what role ANGPTL3 might play. B6:129S5 genetic mice are naturally resistant to nephropathy [26], while C57BL/6 mice are known to be sensitive to drugs that are commonly used to induce nephropathy [26–28]. Therefore, before establishing an animal model of nephropathy in this study, we first used CRISPR/Cas9 technology to knock out the *angptl3* gene in C57BL/6 mice and established *angptl3*-/- mice [12, 13]. Then, we examined the liver and kidney function and structural characteristics of *angptl3*-/- mice in the physiological state. We found that deletion of the *angptl3* gene did not affect kidney morphology or the glomerular and tubular structures of the mice, and no obvious structural abnormalities were observed in podocytes under an electron microscope. No obvious abnormalities in liver cells or bile duct structures in liver tissue were observed by light microscopy. Furthermore, there were no significant changes in serum lipid markers or urine protein levels in *angptl3*-/- mice compared with wild-type mice.

Further analysis of the characteristics of *angptl3* in the nephrosis model confirmed that the proteinuria level of *angptl3*-/- mice after LPS stimulation was significantly lower than that of wild-type mice. In accordance with our previous studies in B6:129S5 genetic mice, the podocytes in mice with *angptl3* gene knockout were slightly fused when viewed under an electron microscope, and the degree of podocyte fusion was significantly reduced compared with that of wild-type mice with nephropathy [11].

Importantly, the hyperlipidemia levels of *angptl3*-/- mice after LPS stimulation were significantly lower than those of wild-type mice. Liver tissue analysis showed that there was significantly less liver cell vacuolization in *angptl3*-/- mice than in wild-type mice after LPS stimulation. Additionally, in vitro data showed that hepatocytes transfected with *angptl3* also appeared with significantly enhanced Oil Red O staining. Compared with wild-type hepatocytes, *angptl3*-knockdown cells showed less staining with Oil Red O after LPS stimulation. Thus far, there has not been an ideal nephrosis animal model similar to human PNS. LPS-treated mice exhibit transient nephrotic syndrome with the pathology of minimal change disease (MCD). Our findings may be applied to other nephrotic models, such as puromycin aminonucleoside (PAN)- or adriamycin-induced focal segmental glomerulosclerosis (FSGS), which are widely accepted as additional nephrosis models.

To demonstrate the role of ANGPTL3 in PNS hyperlipidemia, we also transfected C57 mice with *angptl3* and observed the changes in blood lipids and proteinuria in teg-angptl3 mice at different ages. Our results demonstrated that high ANGPTL3 expression could lead to nephropathic manifestations, such as proteinuria, with increasing age, which suggests that ANGPTL3 participates in the development of both hyperlipidemia and proteinuria in mice. Moreover, it is worthwhile to observe over a longer period to explore possible pathologic changes during the entire life span of teg-angptl3 mice.

In this study, the alterations in LPL in the liver tissue of wild-type or *angptl3*-/- mice in the presence and absence of LPS stimulation suggested that ANGPTL3 markedly inhibited LPL expression in the PNS model. Our experiments showed that a large amount of ANGPTL3 was synthesized by hepatocytes in the context of nephropathy and participated in the occurrence of hyperlipidemia. Based on the reported data, we thinked that some molecules, such as ANGPTL3, played multiple roles not only in proteinuria but also in hyperlipidemia. Our results may also provide new ideas for further study of the pathogenesis of PNS in the future.

## Limitations

This study on ANGPTL3 levels in PNS patients were only a single-center data analysis, and there was no analysis of urine ANGPTL3 expression. Considering the reported population differences in serum ANGPTL3 expression levels, it would be better to expand the data analysis to different regions and nationalities. Our previous studies have shown that podocytes specifically express ANGPTL3, so the expression discrepancies in this molecule in the urine of PNS patients may be more closely related to kidney injury, which required further study. In addition, detection of serum LPL activity in PNS patients or animal models is also necessary to analyze the involvement of ANGPTL3 in the occurrence of nephrotic hyperlipidemia. It should also be noted that the expression of ANGPTL4 and ANGPTL8 in ANGPTL3 knockout mice was not analyzed in this study.

## Conclusions

ANGPTL3 could be involved in the development of dyslipidemia, in addition to proteinuria, during PNS pathogenesis. Inhibiting LPL expression may be the mechanism by which ANGPTL3 induces hyperlipidemia in PNS. Our results suggest that some multifunctional molecules, such as ANGPLT3, are involved in the development of nephrotic syndrome hyperlipidemia and proteinuria. Our study provides new insights into the pathogenesis of nephrotic hyperlipidemia. To date, clinical studies have been carried out using ANGPTL3 inhibitors (specify what and add a reference) to improve the lipid metabolism disorders of patients in the cardiovascular field. These chemicals may also become a potential therapeutic methods for the improvement of nephropathy.

## Supplementary information

**Additional file 1.**

## Data Availability

All data generated or analyzed during this study are included in this published article.

## Abbreviations

PNS: Primary nephrotic syndrome
ANGPTL3: Angiopoietin-like protein 3
angptl3-tg: Angptl3-overexpressing
angptl3-/-: Angptl3-knockout
GAPDH: Glyceraldehyde-phosphate dehydrogenase
DSBs: Double-strand breaks
NHEJ: Nonhomologous end joining
HDR: Homology-directed repair
LPS: Lipopolysaccharide
LXR: Liver X receptors
LPL: Lipoprotein lipase
TG: Triglyceride
TC: Total cholesterol
CHO: Cholesterol
HDL: High-density lipoprotein
LDL: Low-density lipoprotein
Scr: Serum creatinine
BUN: Urea nitrogen
24 hUP: 24-hour urea protein
MCD: Minimal-change disease. WT:wild-type mice
WL: Wild-type mice treated with LPS
KN: Angptl3-/- mice
KL: Angptl3-/- mice stimulated with LPS
Teg: Hepatocytes transfected Angptl3
