# Supplementary material for: ANGPTL3 impacts proteinuria and hyperlipidemia in primary nephrotic syndrome
Source: Lipids Health Dis. 2022 Apr 10;21:38. doi: 10.1186/s12944-022-01632-y (PMC8996604; doi:10.1186/s12944-022-01632-y)
Supplement: Supplementary file 1 — Additional file 1. [file 12944_2022_1632_MOESM1_ESM.zip › supplement table.docx]

**Table S1 Oligonucleotides Used in This Study, Related to Experimental Procedures Oligonucleotides used for making template for in vitro transcription**

| **Template** | **Direction** | **Sequence (5’ to 3’)** |
| --- | --- | --- |
| Cas9 | F | TAATACGACTCACTATAGGGAGAATGGACTATAAGGACCACGAC |
|  | R | GCGAGCTCTAGGAATTCTTAC |
| sgAngptl3 | F | TAATACGACTCACTATAGGTGAAAGGTCTGGATCCACTC |
|  | R | AAAAGCACCGACTCGGTGCC |

**Table S2** **Oligonucleotides used for genotyping of Angptl3^-/-^ mice**

| **Gene target** | **Direction** | **Sequence (5’ to 3’)** |
| --- | --- | --- |
| Angptl3 | F | CATGCAGCAAATAGCCGTGT |
|  | R | CAGGAGGCCATTCGCTAAAA |
