# Supplementary figures and images for: ANGPTL3 impacts proteinuria and hyperlipidemia in primary nephrotic syndrome
Source: Lipids Health Dis. 2022 Apr 10;21:38. doi: 10.1186/s12944-022-01632-y (PMC8996604; doi:10.1186/s12944-022-01632-y)

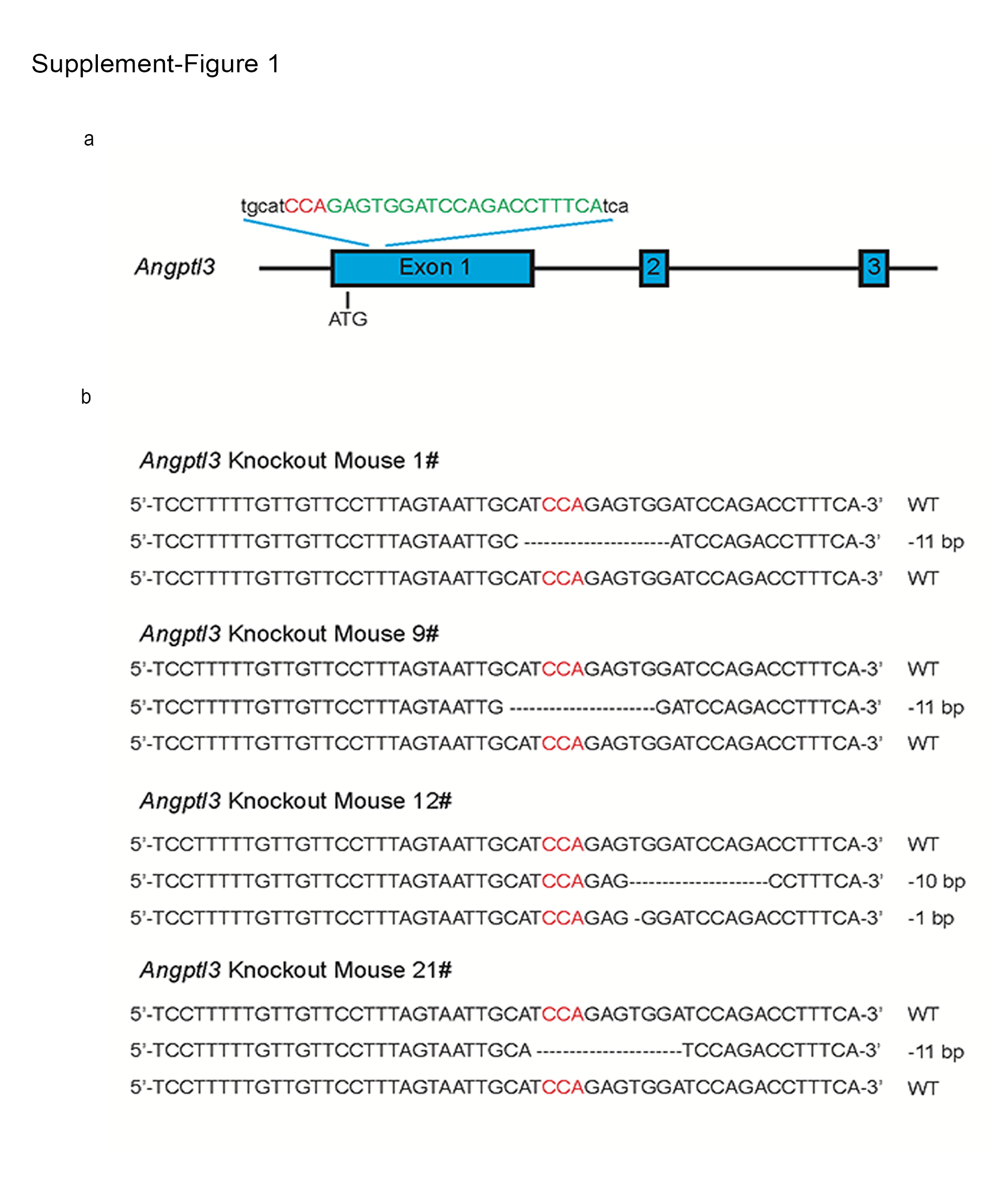

Supplement: Supplementary file 1 — Additional file 1. [file 12944_2022_1632_MOESM1_ESM.zip › supplement-1.tif]

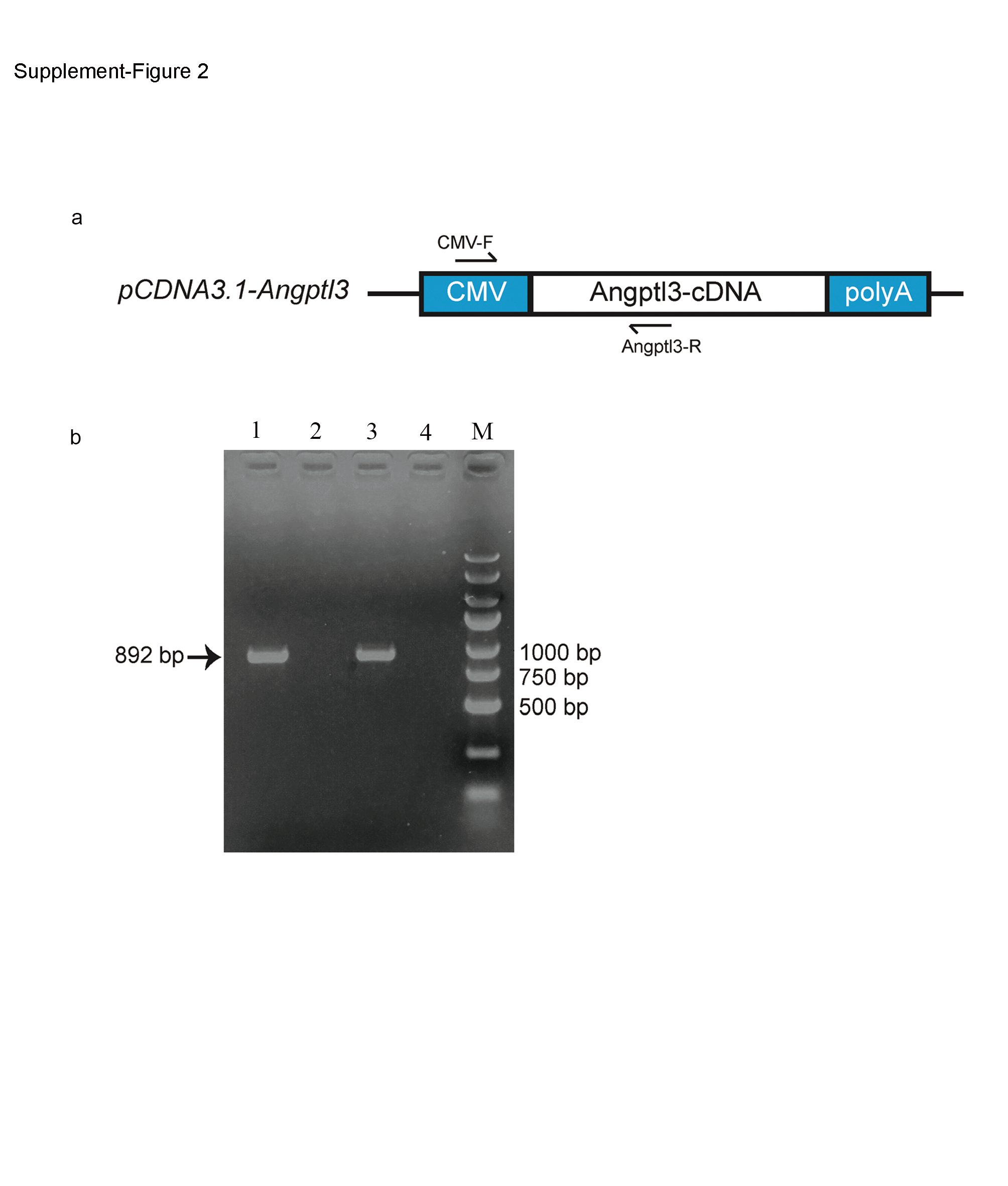

Supplement: Supplementary file 1 — Additional file 1. [file 12944_2022_1632_MOESM1_ESM.zip › supplement-2.tif]
